# Morphological and molecular analyses reveal five new species of *Hydnellum* (*Bankeraceae*, *Thelephorales*) from the Changbai Mountains and Dabie Mountains, China

**DOI:** 10.3897/mycokeys.142.199686

**Published:** 2026-10-15

**Authors:** Yonglan Tuo, Minghao Liu, Zhengxiang Qi, Hang Chu, Jiajun Hu, Bo Zhang, Yu Li, Xiao Li

**Affiliations:** 1 Hefei Mycological Valley Innovation Institute, Hefei, China Hefei Mycological Valley Innovation Institute Hefei China; 2 Key Laboratory of Northern Edible Fungi Resource Utilization, Ministry of Agriculture and Rural Affairs, Jilin Agricultural University, Changchun, 130118, China Key Laboratory of Northern Edible Fungi Resource Utilization, Ministry of Agriculture and Rural Affairs, Jilin Agricultural University Changchun China; 3 College of Life Sciences, Zhejiang Normal University, Jinhua 321000, China College of Life Sciences, Zhejiang Normal University Jinhua China; 4 Sanjiang Fungal Industry Collaborative Innovation Center, Jilin Agricultural University, Changchun 130118, China Sanjiang Fungal Industry Collaborative Innovation Center, Jilin Agricultural University Changchun China; 5 Industrial Development Institute for Plants, Animals and Fungi Integration of Biyang County, Biyang 463799, China Industrial Development Institute for Plants, Animals and Fungi Integration of Biyang County Biyang China

**Keywords:** *
Basidiomycota
*, China, ectomycorrhizal fungi *Hydnellum*, new taxa, phylogeny

## Abstract

This study describes five new *Hydnellum* (*Bankeraceae*, *Thelephorales*) species—*H. aureomarginatum*, *H. coalescens*, *H. flavomarginatum*, *H. imbricatum*, and *H. jianense*—collected from northeastern and central China, based on integrated morphological observations and phylogenetic analyses. Morphologically, *H. aureomarginatum* is characterized by a circular, depressed pileus that bears a golden-yellow zone on its inner margin, with the outermost edge of the pileus remaining white. *Hydnellum coalescens* is characterized by stipes of multiple basidiocarps that are fused or connate (forming a compound or interconnected basidiome) and a pileus covered with brown scales. *Hydnellum flavomarginatum* is characterized by a golden-yellow pileus margin and relatively large basidiospores. *Hydnellum imbricatum* is characterized by layered imbricate scales on the pileus and relatively long basidiospores. *Hydnellum jianense* is characterized by a plano-convex pileus covered with grayish-orange tomentum (which turns light brown when touched) and relatively small basidiospores. This study also compiles statistics on the main morphological characteristics, species distribution, and host information of the *Hydnellum* in China, deepening the understanding of the species diversity and distribution patterns of this genus.

## Introduction

*Hydnellum (Bankeraceae)* is a widely distributed genus of common and important species in *Thelephorales*, established by Karsten (1879). All species within *Hydnellum* are considered ectomycorrhizal (ECM) and are typically found in the soils of natural or less-disturbed forests dominated by *Pinaceae* and *Fagaceae* (Agerer 1993; Arnolds 2010). Morphologically, the genus is characterized by annual basidiomata featuring a zonate or azonate pileal surface, and a spinose hymenophore that varies in color from white to blue, brown, or dark brown (Baird et al. 2013). Microscopically, *Hydnellum* has a monomitic hyphal system with generative hyphae bearing simple septa, and produces yellow to brownish, subglobose to globose, tuberculate basidiospores (Banker 1906; Baird et al. 2013). Traditionally, species identification within *Hydnellum* has relied mainly on macroscopic and microscopic morphological characteristics, such as pileus size, color, and spore dimensions (Banker 1906; Harrison 1964; Baird and Khan 1986). However, many species within the genus exhibit high morphological similarity, and relying solely on morphological features for accurate identification poses significant challenges, likely resulting in an underestimation of the species diversity of this genus (Larsson et al. 2019).

In recent years, researchers have progressively integrated morphological and phylogenetic analyses to advance the taxonomic study of *Hydnellum*. Baird et al. (2013), through their study of stipitate hydnums in the southern United States, recognized 41 taxonomic units, including 19 *Hydnellum* species. Subsequently, Larsson et al. (2019) redefined the generic boundaries between *Hydnellum* and *Sarcodon* based on ITS and nrLSU sequences, transferring 12 species from *Sarcodon* to *Hydnellum* and thereby clarifying the generic delineation. *Hydnellum* has garnered considerable attention in China. For instance, Mu et al. (2021) described 11 new species from China based on morphological and multi-gene phylogenetic analyses. Employing the same approach, Wang et al. (2024) further revised the boundaries between *Sarcodon* and *Hydnellum* and identified three new species within *Hydnellum*. Meanwhile, Song et al. (2022) described five new species of *Hydnellum* from China. In 2025, they further analyzed the molecular phylogenetic relationships and divergence times of *Thelephorales*, described 20 new species, and proposed four new combinations (Song et al. 2025).

Globally, approximately 61 species of the genus *Hydnellum* have been confirmed (Mu et al. 2021), with the majority reported from North America (Banker 1906; Harrison 1964; Baird and Khan 1986; Baird et al. 2013) and Europe (Maas Geesteranus 1975; Kõljalg and Renvall 2000; Loizides et al. 2016). Recent studies in China have significantly contributed to the known diversity of this genus, bringing the number of species documented in China to 48. Among these, over 30 were described during the present decade, with more than half discovered in Sichuan and Yunnan (Mu et al. 2020; Mu et al. 2021; Song et al. 2022; Lei et al. 2023; Wang et al. 2024; Song et al. 2025). These findings highlight southwestern China as a key diversity region for *Hydnellum*, yet vast areas of suitable habitat elsewhere remain underexplored. Moreover, the distribution of *Hydnellum* species is often closely associated with host plants. For example, studies by Arnolds (2003) in the Netherlands demonstrated that local *Hydnellum* species occur mainly in forests dominated by *Pinus sylvestris*, *Quercus robur*, *Quercus rubra*, and *Fagus sylvatica*. Despite the considerable number of *Hydnellum* species reported from China, detailed information on their host tree species remains scarce.

Over the past six years, we have conducted field surveys on macrofungi across northeastern and central China, obtaining an extensive collection of *Hydnellum* specimens. Based on these collections, the present study investigates the diversity of *Hydnellum* species in these regions by integrating morphological observations with phylogenetic analyses of ITS and nrLSU sequences. We describe five new species in detail and provide comprehensive habitat information for these new species—including host plants, climate, and soil properties. We furthermore summarize the distribution and key morphological traits of all known *Hydnellum* species in China. These data are presented to facilitate a better understanding of their ecological habits. Finally, a species identification key is provided to aid in the accurate recognition of these taxa.

## Materials and methods

### Morphological studies

Specimens in this study were collected from the provinces of Anhui and Jilin in China, and their macroscopic characteristics were documented in detail. Subsequently, all samples were dried in a constant-temperature oven at 40–45 °C and deposited in the fungal herbarium of Jilin Agricultural University (**HMJAU**). Colorimetric data were recorded following the Methuen Handbook (Kornerup and Wanscher 1978). Micromorphological analysis was conducted according to our previous method (Tuo et al. 2025). Briefly, sections were mounted in 5% KOH (Sangan Biotech, Shanghai, China, A610441-0500) and examined under a Carl Zeiss Axio Lab A1 compound microscope (Carl Zeiss AG, Jena, Germany) at 1000× magnification. Measurements were taken using an ocular micrometer with an accuracy of 0.1 μm. Amyloid reactions were tested using Melzer’s reagent [3 g KI (Sangan Biotech, Shanghai, China, A610443-0050), 1 g I_2_ (Sangan Biotech, Shanghai, China, A500538-0100), 40 g CCl_3_CH(OH)_2_ (Sangan Biotech, Shanghai, China, A500288-0250), dissolved in 40 mL distilled water]. Spore dimensions (n = 50 per species) were recorded as (a) b–c (d), where ‘a’ and ‘d’ represent the minimum and maximum values, and ‘b–c’ represents the range encompassing 98% of the measurements. Mean length (avg. L) and width (avg. W) were calculated after excluding extreme values; Q denotes the length/width ratio. The following abbreviations and symbols were used: **MR** = Melzer’s reagent (**MR+** = amyloid reaction; **MR–** = nonamyloid); **LCB** = Lactophenol Cotton Blue (**LCB+** = cyanophilous; **LCB–** = acyanophilous) (Yishijiu BIOISCO, Jiangsu, China. SM020-50).

### DNA extraction, PCR, and sequencing

Total genomic DNA was extracted from dried specimens using the NuClear Plant Genomic DNA Kit (CoWin Biotech Co., Ltd., Jiangsu, China, CW0531M) according to the manufacturer’s protocol. The nuclear ribosomal internal transcribed spacer (ITS) region was amplified with primers ITS1F and ITS4 (Mu et al. 2021), and the large subunit of the nuclear ribosomal RNA gene (nrLSU) was amplified with primers LROR and LR7 (Mu et al. 2021). Polymerase chain reaction (PCR) was performed in a 15 μL reaction system containing the following components: 1.0 μL of genomic DNA template; 7.5 μL of SanTaq® PCR Master Mix (Sangon Biotech, Shanghai, China, B532081-0020); 1.0 μL of each forward and reverse primer (10 μM); and 4.5 μL of ddH_2_O. The PCR cycling parameters for the ITS region were as follows: initial denaturation at 95 °C for 3.5 min; 35 cycles of denaturation at 95 °C for 40 s, annealing at 56 °C for 45 s, and extension at 72 °C for 90 s, and a final extension at 72 °C for 10 min. The cycling parameters for nrLSU were: initial denaturation at 94 °C for 2 min; 35 cycles of 94 °C for 40 s, 56 °C for 60 s, and 72 °C for 1.5 min; followed by a final extension at 72 °C for 8 min. The PCR amplicons were purified using a DiaSpin Kit (Sangon Biotech, Shanghai, China, B110093-0100), sent to Comate Bioscience Co., Ltd. (Jilin, China) for sequencing, assembled into consensus sequences using SeqMan (DNASTAR, v7.1), and submitted to GenBank.

### Phylogenetic analyses

A phylogenetic analysis based on the combined ITS and nrLSU dataset was conducted using sequences obtained from pertinent taxonomic studies (Mu et al. 2021; Wang et al. 2024; Song et al. 2025), newly generated sequences from this study, and high-identity BLAST (Basic Local Alignment Search Tool) matches (similarity scores between 97% and 99%) (Table 1). The following species were designated as the outgroup: *Sistotrema muscicola*, *S. leucopus*, *S. scabripes*, and *S. squamosus* (Mu et al. 2021). Sequence alignments for ITS and nrLSU were generated independently using MAFFT v7.505 (Katoh and Standley 2013), manually adjusted in BioEdit v7.1.3 (Hall 1999), and then concatenated using PhyloSuite version 1.2.3 (Xiang et al. 2023). The best-fit partitioning scheme for Bayesian inference and maximum likelihood analyses was selected using ModelFinder v2.2.0 (Kalyaanamoorthy et al. 2017) based on the Bayesian Information Criterion (BIC). Bayesian inference was performed using MrBayes v3.2.7a (Ronquist et al. 2012) with 5 million generations (sampling every 2000 generations, 25% burn-in, split frequency = 0.006, effective sample size (ESS) = 1147.96, potential scale reduction factor (PSRF) = 1.000), and maximum likelihood analysis was performed using IQ-TREE v3.0 (Wong et al. 2025) with 1000 ultrafast bootstraps, as well as the Shimodaira–Hasegawa-like approximate likelihood-ratio test. The final phylogenetic tree was visualized using iTOL v7.2.1 and subsequently edited with Adobe Illustrator 2020 (Adobe, San Jose, CA, USA). In accordance with phylogenetic data standards (Leebens-Mack et al. 2006), the concatenated sequence alignment and associated analysis files have been deposited in the Figshare repository (https://doi.org/10.6084/m9.figshare.32038755.V2).

**Table 1.** Specimens and sequences used in this study.

**Species**	**Country**	**Specimen voucher**	**GenBank No**.		**References**
			**ITS**	**nrLSU**	
*H. ailaoense*	China	HKAS125553 T	OP605523	OP602211	(Lei et al. 2023)
*H. ailaoense*	China	HKAS125554	OP605520	OP602212	(Lei et al. 2023)
*H. amygdaliolens*	Spain	AH 42113 T	JN376763	–	(Larsson et al. 2019)
*H. amygdaliolens*	France	GB-0202072	MW144290	MW144290	(Nitare et al. 2021)
*H. atrorubrum*	China	IFP 019380,	MW579937	–	(Mu et al. 2021)
*H. atrorubrum*	China	IFP 019377 T	MW579936	MW579884	(Mu et al. 2021)
*H. atrospinosum*	China	IFP 018510	MW579940	MW579886	(Mu et al. 2021)
*H. atrospinosum*	China	IFP 018516 T	MW579939	–	(Mu et al. 2021)
*H. aurantiacum*	Norway	EBendiksen177-07	MK602712	MK602712	(Larsson et al. 2019)
*H. aurantiacum*	Sweden	RGCarlsson08-105	MK602711	MK602711	(Larsson et al. 2019)
*H. auratile*	Norway	OF242763	MK602715	MK602715	(Larsson et al. 2019)
*H. auratile*	Norway	OF294095	MK602714	MK602714	(Larsson et al. 2019)
***H. aureomarginatum***	**China**	**FJAU81637**	**PX626420**	**PX623212**	**This study**
***H. aureomarginatum***	**China**	**FJAU81638 T**	**PX626421**	**PX623213**	**This study**
*H. bomiense*	China	IFP 019383	MW579941	MW579887	(Mu et al. 2021)
*H. bomiense*	China	IFP 019382 T	MW579942	–	(Mu et al. 2021)
*H. caeruleum*	Norway	EBendiksen584-11	MK602719	MK602719	(Larsson et al. 2019)
*H. caeruleum*	Norway	EBendiksen575-11	MK602718	MK602718	(Larsson et al. 2019)
*H. caeruleum*	China	IFP 019432	MW579965	–	(Mu et al. 2021)
*H. chocolatum*	China	BJFC 035406 T	ON603657	–	(Song et al. 2022)
*H. chocolatum*	China	BJFC 035404	ON603656	–	(Song et al. 2022)
*H. chrysinum*	–	SC071	KJ534291	–	(Mu et al. 2021)
*H. coactum*	China	IFP 019351 T	MN846278	MN846287	(Mu et al. 2020)
*H. coactum*	China	IFP 019352	MN846279	MN846288	(Mu et al. 2020)
*H. complicatum*	USA	REB-71	KC571711	–	(Baird et al. 2013)
*H. complicatum*	USA	REB-329	KC571712	–	(Baird et al. 2013)
*H. concentricum*	China	BJFC 030316 T	ON603658	ON603639	(Song et al. 2022)
*H. concrescens*	USA	SEW 88	AY569025	–	(Mu et al. 2021)
*H. concrescens*	Mexico	GO-2009-204	KC152116	–	(Mu et al. 2021)
***H. coalescens***	**China**	**FJAU81635 T**	**PX626425**	**PX623218**	**This study**
***H. coalescens***	**China**	**FJAU81636**	**PX626426**	**PX623219**	**This study**
*H. crassipileatum*	China	BJFC 030318	ON603659	ON603642	(Song et al. 2022)
*H. crassipileatum*	China	BJFC 030320 T	ON603660	ON603641	(Song et al. 2022)
*H. cristatum*	USA	REB-169	JN135174	–	(Baird et al. 2013)
*H. cristatum*	USA	REB-88	KC571718	–	(Baird et al. 2013)
*H. dianthifolium*	Cyprus	ML61211HY T	KX619419	–	(Loizides et al. 2016)
*H. dianthifolium*	Cyprus	ML902162HY	KX619420	–	(Loizides et al. 2016)
*H. earlianum*	USA	REB-75	KC571724	–	(Baird et al. 2013)
*H. earlianum*	USA	REB-375	JN135179	JN135179	(Baird et al. 2013)
*H. edulium*	China	SAAS 2870	OK636094	OP407675	(Wang et al. 2024)
*H. edulium*	China	SAAS 2920	OK636092	OP407677	(Wang et al. 2024)
*H. fagiscabrosum*	Sweden	GB-0195805 T	MW144294	MW144294	(Nitare et al. 2021)
*H. fagiscabrosum*	Sweden	GB-0195623	MW144295	MW144295	(Nitare et al. 2021)
*H. fennicum*	Norway	O-F-242833	MK602738	MK602738	(Larsson et al. 2019)
*H. fennicum*	Norway	O-F-294087	MK602737	MK602737	(Larsson et al. 2019)
*H. ferrugineum*	Sweden	E Larsson 197-14	MK602722	MK602722	(Larsson et al. 2019)
*H. ferrugineum*	Sweden	E Larsson 356-16	MK602721	MK602721	(Larsson et al. 2019)
*H. ferrugipes*	USA	REB-176	KC571727	–	(Baird et al. 2013)
*H. ferrugipes*	USA	REB-58	JN135176	–	(Baird et al. 2013)
***H. flavomarginatum***	**China**	**FJAU81640**	**PX626416**	**PX623209**	**This study**
***H. flavomarginatum***	**China**	**FJAU81641**	**PX626419**	**PX623210**	**This study**
***H. flavomarginatum***	**China**	**FJAU81639 T**	**PX626424**	**PX623211**	**This study**
*H. fuligineoviolaceum*	Sweden	B Nylen 130918	MK602741	MK602741	(Larsson et al. 2019)
*H. fuligineoviolaceum*	Sweden	LA 120818	MK602740	MK602740	(Larsson et al. 2019)
*H. fuscoindicum*	USA	OSC 107844	EU669229	EU669279	(Larsson et al. 2019)
*H. fuscoindicum*	USA	OSC 113622	EU669228	EU669278	(Larsson et al. 2019)
*H. geogenium*	USA	AFTOL-ID 680	DQ218304	AY631900	(Matheny et al. 2007)
*H. geogenium*	Norway	O-F-296213	MK602724	MK602724	(Larsson et al. 2019)
*H. glaucopus*	Sweden	UPS-F013955 T	MW144330	MW144330	(Nitare et al. 2021)
*H. glaucopus*	Sweden	GB-0195644	MW144319	MW144319	(Nitare et al. 2021)
*H. gracilipes*	Sweden	E Larsson 219-11	MK602726	MK602726	(Larsson et al. 2019)
*H. gracilipes*	Sweden	GB-0113779	MK602727	MK602727	(Larsson et al. 2019)
*H. grosselepidotum*	China	IFP 019355	MN846274	MN846283	(Mu et al. 2020)
*H. grosselepidotum*	China	IFP 019353	MN846276	MN846285	(Mu et al. 2020)
*H. illudens*	Sweden	GB-0195819	MW144341	MW144341	(Nitare et al. 2021)
*H. illudens*	Norway	O-F-242769	MW144335	MW144335	(Nitare et al. 2021)
***H. imbricatum***	**China**	**FJAU81633 T**	**PX626422**	**PX623214**	**This study**
***H. imbricatum***	**China**	**FJAU81634**	**PX626423**	**PX623215**	**This study**
*H. inflatum*	China	IFP 019404	MW579949	MW579894	(Mu et al. 2021)
*H. inflatum*	China	IFP 019403 T	MW579950	MW579895	(Mu et al. 2021)
***H. jianense***	**China**	**FJAU81642 T**	**PX626417**	**PX623216**	**This study**
***H. jianense***	**China**	**FJAU81643**	**PX626418**	**PX623217**	**This study**
*H. joeides*	Sweden	K Hjortstam 17589	MK602750	MK602750	(Larsson et al. 2019)
*H. joeides*	Sweden	J Nitare 110829	MK602751	MK602751	(Larsson et al. 2019)
*H. lepidus*	Sweden	J Nitare 110829	MK602754	MK602754	(Larsson et al. 2019)
*H. lepidus*	Sweden	RG Carlsson 10-065	MK602752	MK602752	(Larsson et al. 2019)
*H. lidongensis*	China	IFP 019357 T	MN846280	MN846289	(Mu et al. 2020)
*H. lidongensis*	China	IFP 019358	MN846281	MN846290	(Mu et al. 2020)
*H. lundellii*	Norway	O-F-242639	MK602750	MK602750	(Larsson et al. 2019)
*H. lundellii*	Norway	O-F-295814	MK602751	MK602751	(Larsson et al. 2019)
*H. martioflavum*	Norway	O-F-242435	MK602754	MK602754	(Larsson et al. 2019)
*H. martioflavum*	Norway	O-F-242872	MK602752	MK602752	(Larsson et al. 2019)
*H. melanocarpum*	China	BJFC 035418	ON603662	ON603643	(Song et al. 2022)
*H. melanocarpum*	China	BJFC 035420	ON603663	ON603644	(Song et al. 2022)
*H. mirabile*	Sweden	S Lund 140912	MK602730	MK602730	(Larsson et al. 2019)
*H. mirabile*	Sweden	E Larsson 170-14	MK602729	MK602729	(Larsson et al. 2019)
*H. nemorosum*	Norway	O-F-242352	MW144372	MW144372	(Nitare et al. 2021)
*H. nemorosum*	Sweden	GB-0195631	MW144373	MW144373	(Nitare et al. 2021)
*H. parvum*	USA	REB-131	JN135187	–	(Baird et al. 2013)
*H. parvum*	USA	REB-392	KC571717	–	(Baird et al. 2013)
*H. peckii*	China	IFP 0193433	MW579966	MW579905	(Mu et al. 2021)
*H. peckii*	China	IFP 019434	MW579967	MW579906	(Mu et al. 2021)
*H. pineticola*	USA	REB-49	KC571733	–	(Baird et al. 2013)
*H. piperatum*	USA	REB-322	JN135173	–	(Baird et al. 2013)
*H. piperatum*	USA	REB-304	KC571723	–	(Baird et al. 2013)
*H. roseoviolaceum*	Sweden	GB-0195936 T	NR185593	MW144374	(Nitare et al. 2021)
*H. roseoviolaceum*	Sweden	GB-0195687	MW144375	MW144375	(Nitare et al. 2021)
*H. rubidofuscum*	China	IFP 019408 T	MW579951	MW579896	(Mu et al. 2021)
*H. rubidofuscum*	China	IFP 019417	MW579953	MW579898	(Mu et al. 2021)
*H. scabrosellum*	Sweden	GB-0195792	MW144380	MW144380	(Nitare et al. 2021)
*H. scabrosellum*	Sweden	GB-0195689 T	MW144379	MW144379	(Nitare et al. 2021)
*H. scabrosum*	Norway	O-F-360777	MK602765	MK602765	(Larsson et al. 2019)
*H. scabrosum*	Norway	O-F-292320	MK602766	MK602766	(Larsson et al. 2019)
*H. scleropodium*	USA	REB-3	JN135186	–	(Baird et al. 2013)
*H. scleropodium*	USA	REB-352	KC571740	–	(Baird et al. 2013)
*H. scrobiculatum*	USA	REB-78	JN135181	–	(Baird et al. 2013)
*H.* sp. 4	China	IFP 019439	MW579972	–	(Mu et al. 2021)
*H. spongiosipes*	China	IFP 019435	MW579968	MW579907	(Mu et al. 2021)
*H. squamulosum*	China	IFP 019418 T	MW579954	–	(Mu et al. 2021)
*H. squamulosum*	China	IFP 019420	MW579956	MW579899	(Mu et al. 2021)
*H. suaveolens*	Sweden	E Larsson 8-14	MK602735	MK602735	(Larsson et al. 2019)
*H. suaveolens*	Norway	S Svantesson 877	MK602736	MK602736	(Larsson et al. 2019)
*H. subalpinum*	China	SAAS 2778 T	OP437919	OP407685	(Wang et al. 2024)
*H. subalpinum*	China	SAAS 2884	OP437920	OP407686	(Wang et al. 2024)
*H. subsuccosum*	USA	SEW-55	AY569033	–	(Mu et al. 2021)
*H. subsuccosum*	USA	REB-10	JN135178	–	(Baird et al. 2013)
*H. underwoodii*	USA	REB-358	JN135189	–	(Baird et al. 2013)
*H. underwoodii*	USA	REB-119	KC571782	–	(Baird et al. 2013)
*H. versipelle*	China	SAAS 2905	OP437924	OP407690	(Wang et al. 2024)
*H. versipelle*	China	SAAS 2841	OP437923	OP407689	(Wang et al. 2024)
*H. yunnanense*	China	IFP 019429 T	MW579962	MW579903	(Mu et al. 2021)
*H. yunnanense*	China	IFP 019430	MW579963	MW579904	(Mu et al. 2021)
*S. leucopus*	Norway	O-F-296099	MK602755	MK602755	(Mu et al. 2021)
*S. leucopus*	Sweden	P Hedberg 080811	MK602757	MK602757	(Larsson et al. 2019)
*S. scabripes*	Mexico	FCME:23240	EU293829	–	(Mu et al. 2021)
*S. scabripes*	USA	REB-351	JN135191	–	(Baird et al. 2013)
*S. squamosus*	Norway	O-F-177452	MK602768	MK602768	(Larsson et al. 2019)
*S. squamosus*	Norway	O-F-295554	MK602769	MK602769	(Larsson et al. 2019)

Note: Newly generated sequences in this study are shown in bold. Type specimens are marked with a T.

## Results

### Phylogenetic analyses

**Figure 1.**
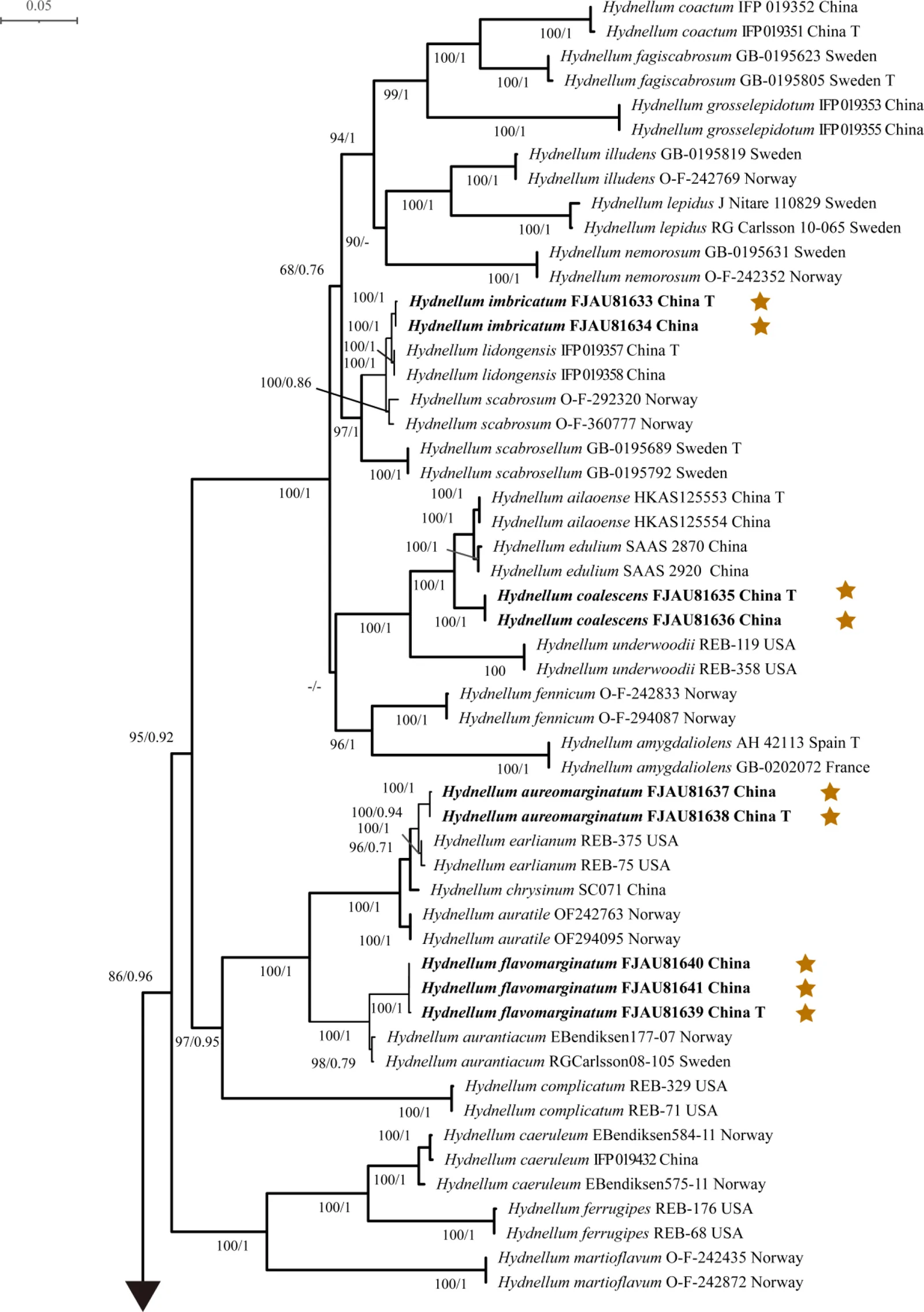
The phylogram of *Hydnellum* inferred from Bayesian analyses using ITS + nrLSU dataset. The values on the nodes represent Bayesian posterior probability (left, BPP ≥ 0.95) and maximum likelihood bootstrap support (right, MLBS ≥ 75%), respectively. Holotype specimens are marked with T. New taxa are marked with brown stars.

**Figure 2.**
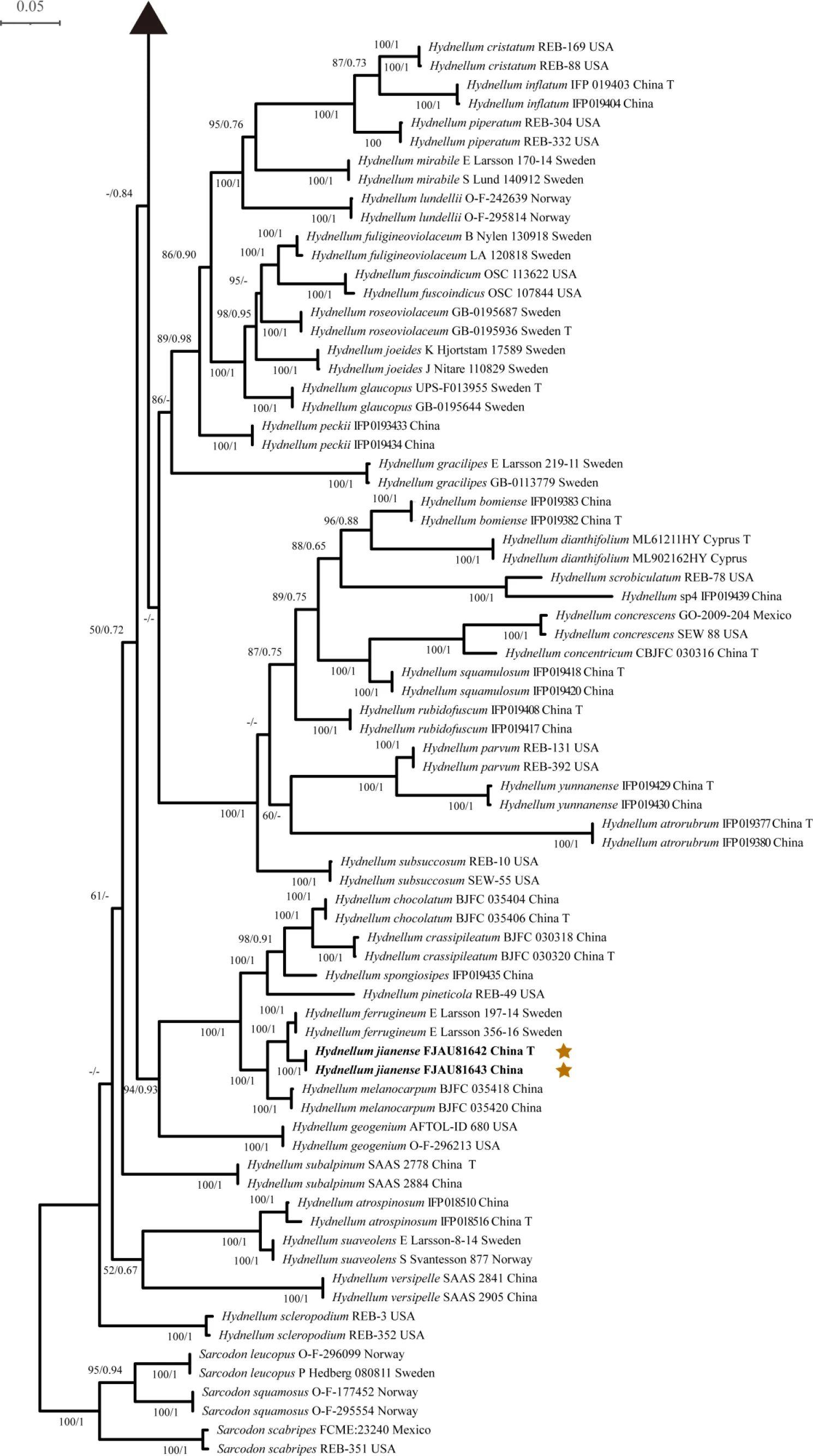
The phylogram of *Hydnellum* inferred from Bayesian analyses using ITS + nrLSU dataset. The values on the nodes represent Bayesian posterior probability (left, BPP ≥ 0.95) and maximum likelihood bootstrap support (right, MLBS ≥ 75%), respectively. Holotype specimens are marked with T. New taxa are marked with brown stars.

The combined ITS + nrLSU dataset comprised 221 sequences from 128 specimens representing 66 taxa. The final dataset had an aligned length of 2604 sites, containing 1601 constant sites (61.48%), 87 singleton sites (3.34%), and 915 parsimony-informative sites (35.14%). In both the maximum likelihood (ML) phylogenetic analysis and Bayesian inference (BI) analysis, the best-fit partitioned models were selected using the Bayesian Information Criterion (BIC). Specifically, for the maximum likelihood (ML) analysis, the best-fit partitioned models were TIM3+F+I+G4 for ITS, GTR+F+I+R2 for nrLSU. Similarly, for the Bayesian inference (BI) analysis, the best-fit partitioned models were GTR+F+I+G4 for ITS, SYM+I+G4 for nrLSU. There was topological consistency between the maximum likelihood (ML) and Bayesian inference (BI) methods, and the ML tree is provided in this study (Figs 1, 2).

The newly generated sequences were divided into five well-supported (BPP/MLBS = 1/100) species-level clades. Specifically, *H. coalescens* (BPP/MLBS = 1/100) and *H. flavomarginatum* (BPP/MLBS = 1/100) formed robust independent lineages, while *H. aureomarginatum* (BPP/MLBS = 1/100), *H. imbricatum* (BPP/MLBS = 1/100), and *H. jianense* (BPP/MLBS = 1/100) each formed a sister-group relationship with *H. earlianum*, *H. lidongensis*, and *H. ferrugineum*, respectively (i.e., *H. aureomarginatum* with *H. earlianum*, etc.) (Figs 1, 2).

### Taxonomy

#### Hydnellum aureomarginatum

Taxon classificationFungiThelephoralesBankeraceae

Yonglan Tuo, Bo Zhang & Yu Li sp. nov.

6428AF5F-3FC7-50C9-9E66-19C350F54148

Fungal Names: FN 573075

Fig. 3A–G

##### Etymology.

The specific epithet “*aureomarginatum*” refers to the golden yellow zone on the inner side of the pileus margin, while the outermost edge remains white.

##### Holotype.

China • Jilin Province, Ji’an City, Jian National Nature Reserve, 41°16'22"N, 126°7'23"E, Elevation: ca. 736 m, on the ground in a *Quercus mongolica* Fisch. ex Ledeb. forest, 14 Aug 2019, Yonglan Tuo FJAU81637. GenBank numbers: ITS = PX626420, nrLSU = PX623212.

##### Diagnosis.

*H. aureomarginatum* is distinguished from other *Hydnellum* species by its deeply infundibuliform pileus with a central opening, pubescent surface, and a golden-yellow zone on the inner margin (the extreme edge remaining white), as well as short, decurrent spines. Microscopically, it exhibits subglobose to ellipsoid basidiospores ornamented with tuberculis that are predominantly isolated or paired. Hyphae are uniformly ≤ 5 µm wide and lack clamp connections. Fruiting in autumn in stands of *Q. mongolica*.

**Figure 3.**
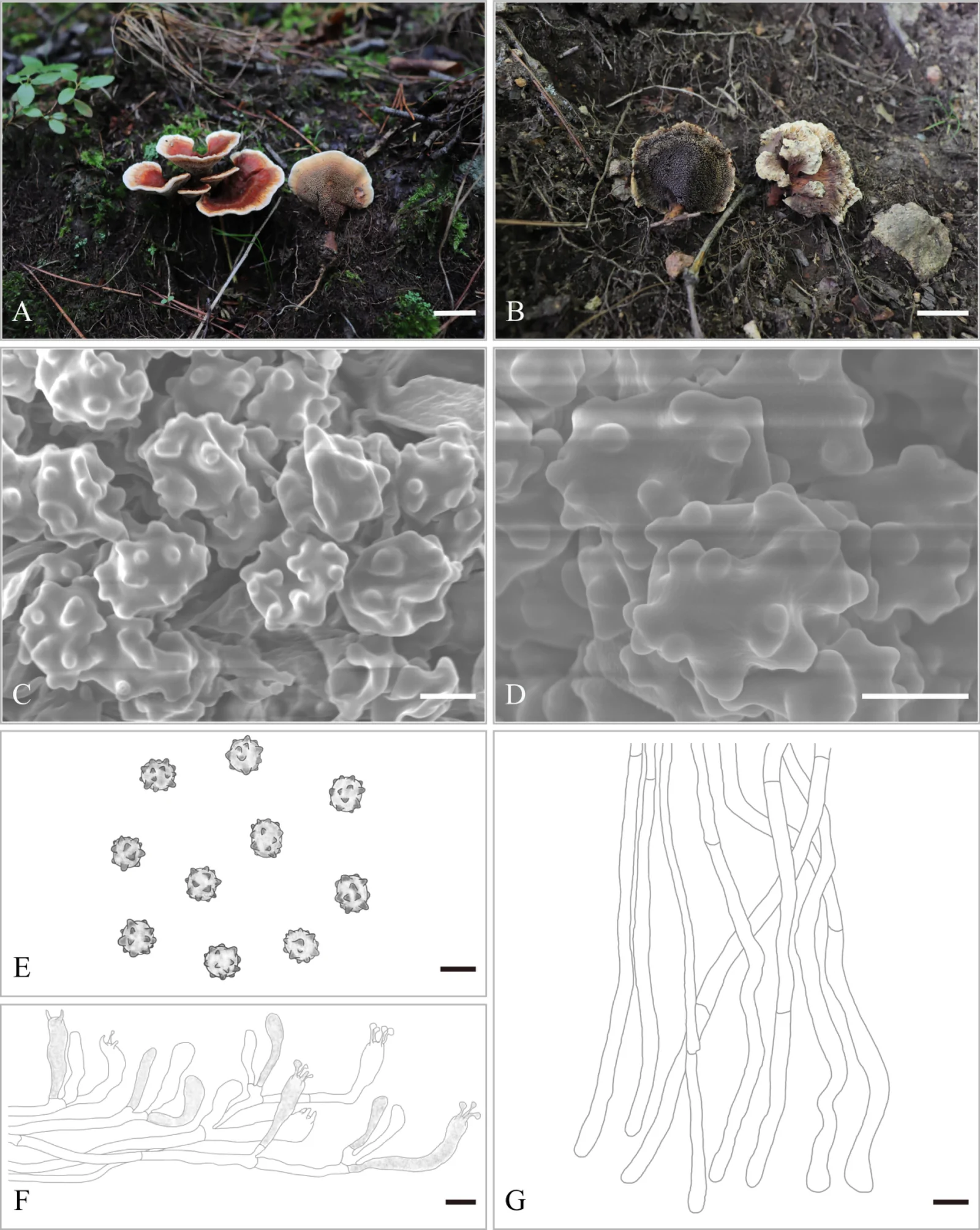
*Hydnellum aureomarginatum*. **A, B**. Basidiocarps; **C, D**. SEM of basidiospores (SEM images from FJAU81637); **E–G**. Microscopic structures (drawn from FJAU81637); **E**. Basidiospores; **F**. Hymenium and subhymenium; **G**. Pileipellis. Scale bars: 2 cm (**A, B)**; 2.5 μm (**C, D**); 5 μm (**E**); 10 μm (**F, G**).

##### Description.

Basidiocarps small to medium, gregarious, 35.5–50.6 mm in height. Pileus 17.6–42.3 mm wide, irregularly circular to flabelliform, depressed centrally, brownish orange (6C8) to dark brown (6F8), zonate; surface pubescent to floccose when fresh, becoming matted or fibrillose when dry; inner margin golden yellow (5B7), and outer margin incurved and covered with white (4A1) tomentum. Pileus context 1.0–2.2 mm thick, deep orange (6A6). Spines decurrent, light orange (7D8) to light brown (6D5) when fresh, grayish brown (6F3) when dry, slightly sparse, 1–2 spines/mm^2^, 0.5–2.5 mm long, 0.1–0.5 mm in diameter. Stipe 11.3–20.5 × 3.0–10.2 mm, dark brown (6A6), usually eccentric, cylindrical and somewhat inflated at the base, solid. Odor mild or fruity.

Basidiospores (4.0) 5.0–5.5 (6.0) × 4.0–5.0 μm, av. L = 5.01 μm, av. W = 4.72 μm, Q = 1.00–1.25 (1.5), av. Q = 1.06, subglobose to ellipsoid, light brown, thin-walled, tuberculate, MR+, LCB–; tuberculi usually isolated or grouped in two, 0.3–0.8 μm long, 0.5–0.6 μm in diameter. Basidia 30.0–35.0 × 6.0–7.0 μm, clavate to suburniform, some with granular contents; sterigmata 2–4, 2.5–4.0 × 0.5–1.0 µm. Basidioles 15.5–22.5 × 5.0–6.0 μm, smaller than basidia. Subhymenium trama filamentous, hyphae 3.5–4.0 μm wide, septate, thin-walled, hyaline in 5% KOH. Hyphae of pileus context 4.5–5.0 μm, thin-walled. Pileipellis composed of cylindrical hyphae, slightly interwoven, rarely branched, terminal elements cylindrical at apex, cells 38.5–120.5 × 4.0–5.0 μm. Hyphae from the inner layer of stipe, 3.5–4.0 μm wide, interwoven, occasionally branched. Hyphae from the surface layer of stipe, 3.0–3.5 μm wide, subparallel, occasionally branched. Clamp connections not observed.

##### Habitat and distribution.

This species was observed growing solitarily in a *Q. mongolica* forest within the buffer zone of Jian National Nature Reserve, Jilin, China (ca. 700–850 m, north temperate continental climate), an area with minimal human disturbance. Two specimens were collected 205.6 cm and 62.5 cm from the base of a *Q. mongolica* tree (diameter at breast height [DBH] = 23.7 cm and 23.2 cm, respectively). At the time of collection, the environmental parameters for the two specimens were as follows: soil temperature was 22.2 °C and 21.9 °C; volumetric soil water content was 18.8% and 20.6%; soil pH was 5.1 and 5.3; air temperature was 24.7 °C and 24.8 °C; relative humidity of 82.2% and 83.9%.

##### Additional specimens examined.

China • Jilin Province, Ji’an City, Jian National Nature Reserve, 41°16'29"N, 126°7'20"E, Elevation: ca. 805 m, on soil in a *Q. mongolica* forest, 17 Sep 2019, Yonglan Tuo FJAU81638. GenBank numbers: ITS = PX626421, nrLSU = PX623213.

##### Notes.

*Hydnellum aureomarginatum* resembles *H. earlianum* in possessing a zonate pileus, decurrent spines, and tuberculate basidiospores, but is readily distinguished by the following suite of characters:

Macromorphologically, the pileus of *H. aureomarginatum* is considerably smaller (17.6–42.3 mm) than that of *H. earlianum* (up to 90 mm). Crucially, the pileus margin comprises a golden-yellow, zonate inner margin and a persistently tomentose, white (4A1) outer margin—a condition absent in *H. earlianum*, which exhibits a broader color range (pale yellow to copper) and becomes glabrous upon drying. Furthermore, *H. aureomarginatum* bears notably shorter (0.5–2.5 mm), sparser spines (1–2 per mm^2^), contrasting with the crowded, longer spines (up to 4.0 mm) of *H. earlianum*.

Ecologically, *H. aureomarginatum* occurs in *Q. mongolica* forests in the eastern mountainous region of Jilin Province, Northeast China. In contrast, *H. earlianum* occurs in temperate mixed forests of the southern Appalachian Mountains, where it associates with *Acer rubrum*, *Betula alleghaniensis*, *Carya glabra*, *Fagus grandifolia*, *Tsuga canadensis*, and *Q. velutina*.

Micromorphologically, basidiospores of *H. aureomarginatum* are larger in size (mean 5.01 × 4.72 μm) than those of *H. earlianum* (mean 4.51 × 3.65 μm). The ornamentation differs markedly: tubercles in *H. aureomarginatum* are typically isolated or paired, whereas those in *H. earlianum* are flattened to exsculptate and irregularly arranged. Hyphae of *H. aureomarginatum* are uniformly ≤ 5 μm wide. In contrast, *H. earlianum* possesses wider hyphae in the pileus trama (up to 6.5 μm) and stipe hyphae (up to 5.0 μm), in addition to distinctive gloeopleurous-like hyphae (up to 7.0 μm).

Phylogenetically, *H. aureomarginatum* and *H. earlianum* form two independent, strongly supported clades in the concatenated ITS + nrLSU phylogeny (MLBS = 100%, BPP = 1.0), further corroborating their status as distinct species.

#### Hydnellum coalescens

Taxon classificationFungiThelephoralesBankeraceae

Yonglan Tuo, Bo Zhang & Yu Li sp. nov.

E0204C76-C5C0-5ADF-8C01-EB4170559398

Fungal Names: FN 573074

Fig. 4A–G

##### Etymology.

The specific epithet “*coalescens*” refers to the fused or connate stipes of multiple fruiting bodies, which form a compound or interconnected basidiome.

##### Holotype.

China • Anhui Province, Lu’an City, Tianma National Nature Reserve, 31°18'58"N, 115°55'32"E, Elevation: ca. 805 m, on soil in a forest of *Quercus serrata* Thunb. forest, 11 Oct 2023, Yonglan Tuo FJAU81635. GenBank numbers: ITS = PX626425, nrLSU = PX623218.

##### Diagnosis.

*H. coalescens* is distinguished by stipes of multiple basidiomes fused or connate into a compound basidiome. Basidiocarps medium to large; pileus yellowish-brown, flabelliform, with irregularly arranged squamules; spines white, deeply decurrent to the stipe base; basidiospores globose to subglobose, isodiametric, 5.0–6.0 × 5.0–6.0 μm, ornamented with irregular, acute tubercles typically clustered in 2–3-membered groups. Occurring solitary to gregarious in autumn in *Q. serrata* forests.

**Figure 4.**
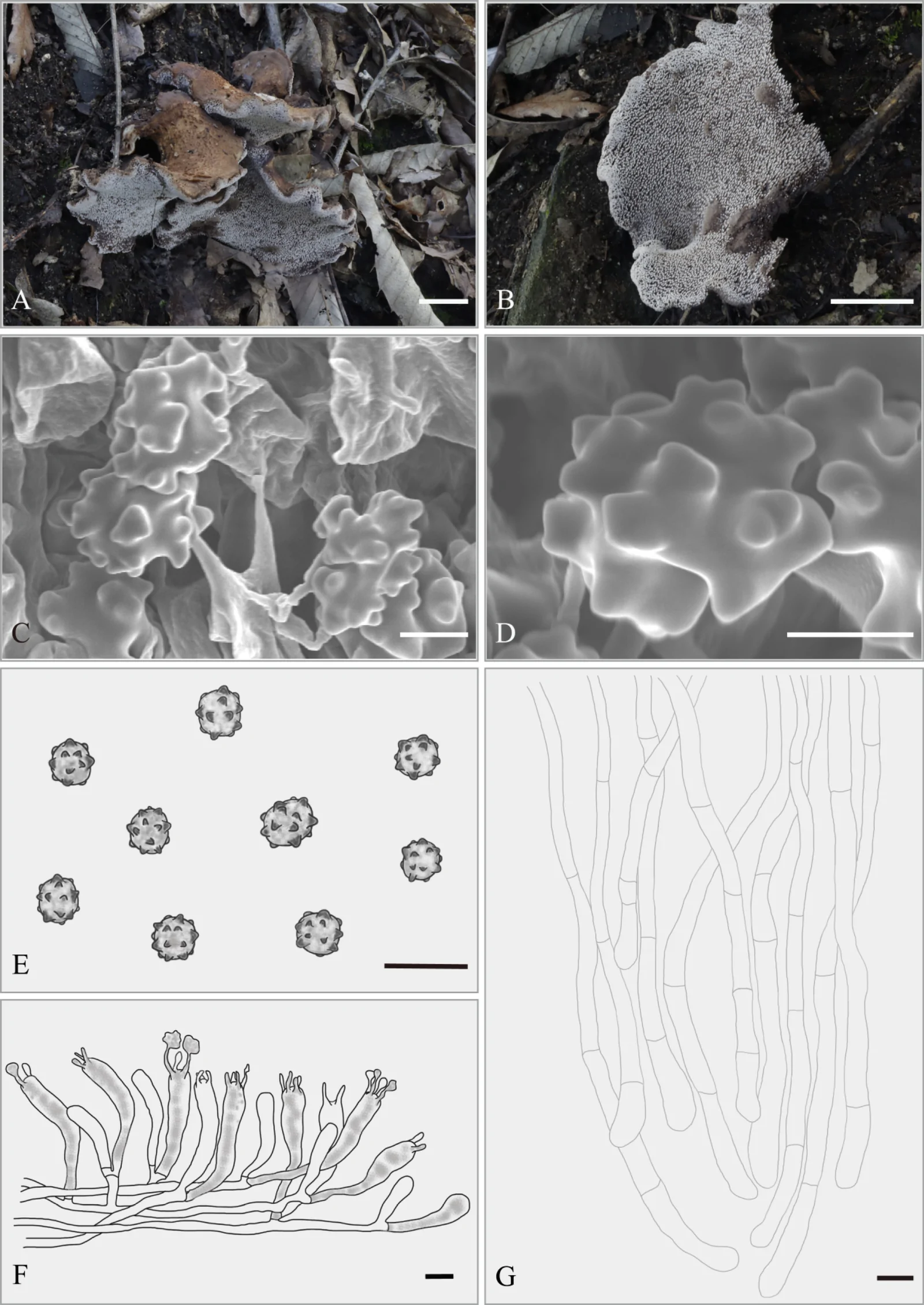
*Hydnellum coalescens*. **A, B**. Basidiocarps; **C, D**. SEM of basidiospores (SEM images from FJAU81635); **E–G**. Microscopic structures (drawn from FJAU81635); **E**. Basidiospores; **F**. Hymenium and subhymenium; **G**. Pileipellis. Scale bars: 2 cm (**A, B**); 2.5 μm (**C, D**); 10 μm (**E–G**).

##### Description.

Basidiocarps medium to large, 40.0–53.6 mm high, solitary to gregarious, fleshy when fresh. Pileus 63.6–80.5 mm wide, azonate, flabelliform; surface covered with irregularly arranged, brownish yellow (5C7) scales; Margin recurved and covered with white (4A1) tomentum. Spines decurrent, white to yellowish white (4A1–4A2), slightly sparse, 1–2 spines/mm^2^, 2.0–6.2 mm long, 0.1–0.5 mm in diameter. Stipe 30.0–35.5 × 15.0–23.0 mm, solid, cylindrical, eccentric to central, covered by short spines, and stipes fused or connate to form a compound cluster. Odor mild or fruity.

Basidiospores 5.0–6.0 × 5.0–6.0 μm, av. L = 5.29 μm, av. W = 5.19 μm, Q = 1.00–1.10 (1.20), av. Q = 1.02, globose to subglobose, brown, thin-walled, tuberculate, MR–, LCB+; tuberculi usually grouped in 2–3, 0.5–0.8 μm long, 0.5–1.0 μm in diameter. Basidia (25.5) 30.0–40.0 × 6.0–7.0 μm, clavate to suburniform, some with granular contents; sterigmata 2–4, 3.0–4.5 × 0.5–1.0 µm, conical. Basidioles 20.0–22.5 × 6.0–7.0 μm, smaller than basidia. Subhymenium trama filamentous, hyphae 3.0–3.5 μm wide, septate, thin-walled, hyaline in 5% KOH. Hyphae of pileus context 7.5–9.0 μm, thin-walled. Pileipellis composed of cylindrical hyphae, slightly interwoven, rarely branched, terminal elements cylindrical at apex, cells 20.0–60.5 × 5.0–7.0 μm. Hyphae from the inner layer of stipe, 9.0–10.0 μm wide, subparallel, occasionally branched. Hyphae from the surface layer of stipe, 5.5–7.0 μm wide, slightly interwoven, occasionally branched. Clamp connections not observed.

##### Habitat and distribution.

This species was observed growing solitarily in a *Q. serrata* forest in the buffer zone of Tianma National Nature Reserve, China (ca. 800–900 m; subtropical monsoon climate). The site experiences anthropogenic disturbances, including grazing and selective thinning, and is located 0.5–4.2 km from the nearest settlements. Two specimens were collected 65.2 cm (DBH = 13.5 cm) and 80.3 cm (DBH = 16.8 cm) from *Q. serrata* trees. At the time of collection, the environmental parameters for the two specimens were as follows: soil temperature was 18.8 °C and 19.2 °C; soil volumetric water content was 15.6% and 14.1%; soil pH was 5.3 and 5.4; air temperature was 23.1 °C and 22.8 °C; relative humidity was 88.2% and 87.5%, respectively.

##### Additional specimens examined.

China • Anhui Province, Lu’an City, Tianma National Nature Reserve, 31°18'56"N, 115°55'33"E, Elevation: ca. 813 m, on the ground in a *Q. serrata* forest, 11 Oct 2023, Yonglan Tuo FJAU81636. GenBank numbers: ITS = PX626426, nrLSU = PX623219.

##### Notes.

Macromorphologically, *H. coalescens* resembles *H. edulium* in pileus size, coloration, and decurrent spines. However, it differs in its flabelliform pileus, whereas that of *H. edulium* is applanate to centrally depressed (subcircular). Additionally, *H. coalescens* bears squamules that are comparatively smaller than the conspicuous, coarse scales typical of *H. edulium*. Crucially, *H. coalescens* is distinguished by its compound basidiome formed by confluent stipes—a condition absent in *H. edulium*, which produces solitary, simple basidiomes. Furthermore, *H. coalescens* lacks the distinctive bluish-green stipe base characteristic of *H. edulium*.

Micromorphologically, basidiospores of *H. coalescens* are globose to subglobose (5.0–6.0 × 5.0–6.0 μm), ornamented with regular, acutely tipped tubercles occurring singly or in clusters of 2–3. By contrast, *H. edulium* produces narrowly ellipsoid to irregular basidiospores (4.5–5.5 × 5.5–7.0 μm) with flattened to exsculptate, more complex ornamentation. Critically, the hymenophoral tramal hyphae (up to 7 μm wide) and pileipellis terminal cells (up to 11–20 μm wide) of *H. edulium* are markedly broader than the corresponding structures in *H. coalescens* (tramal hyphae 3.0–3.5 μm wide; terminal cells 5.0–7.0 μm wide), and contain conspicuous brownish intracellular pigments—features not observed in *H. coalescens*.

Ecologically, *H. coalescens* typically fruits gregariously in montane *Q. serrata* forests of central China (Anhui Province). In contrast, *H. edulium* occurs in southwestern China (Sichuan Province), typically as solitary basidiocarps in forests dominated by *Pinus* and *Quercus*.

Phylogenetic analyses of the concatenated ITS + nrLSU dataset (Fig. 1) further confirm that *H. coalescens* and *H. edulium* resolve into two independent, strongly supported clades (MLBS = 100%, BPP = 1.0), corroborating their recognition as distinct species.

#### Hydnellum flavomarginatum

Taxon classificationFungiThelephoralesBankeraceae

Yonglan Tuo, Bo Zhang & Yu Li sp. nov.

F956DEAA-9233-559D-B3FE-DE7379348CE4

Fungal Names: FN 573076

Fig. 5A–I

##### Etymology.

The specific epithet “*flavomarginatum*” reflects the characteristic yellow margin of the pileus.

##### Holotype.

China • Jilin Province, Ji’an City, Jian National Nature Reserve, 41°16'21"N, 126°7'10"E, Elevation: ca. 695 m, on soil in a forest of *Quercus mongolica* Fisch. ex Ledeb. forest, 14 Sep 2020, Yonglan Tuo FJAU81639. GenBank numbers: ITS = PX626424, nrLSU = PX623211.

##### Diagnosis.

*H. flavomarginatum* is readily distinguished from other *Hydnellum* congeners by its persistently golden-yellow pileus margin, retained from youth to maturity. Basidiocarps small to medium; pileus brownish-orange to dark brown, circular to subcircular, centrally depressed, with conspicuous concentric zonation; spines subdecurrent, white to grayish-brown; basidiospores globose to subglobose (6.0–7.0 × 6.0–6.5 μm), with isolated or paired tubercles; clamp connections occasionally present in the pileipellis. Occurring solitary to gregarious in *Q. mongolica* forests of Northeast China.

**Figure 5.**
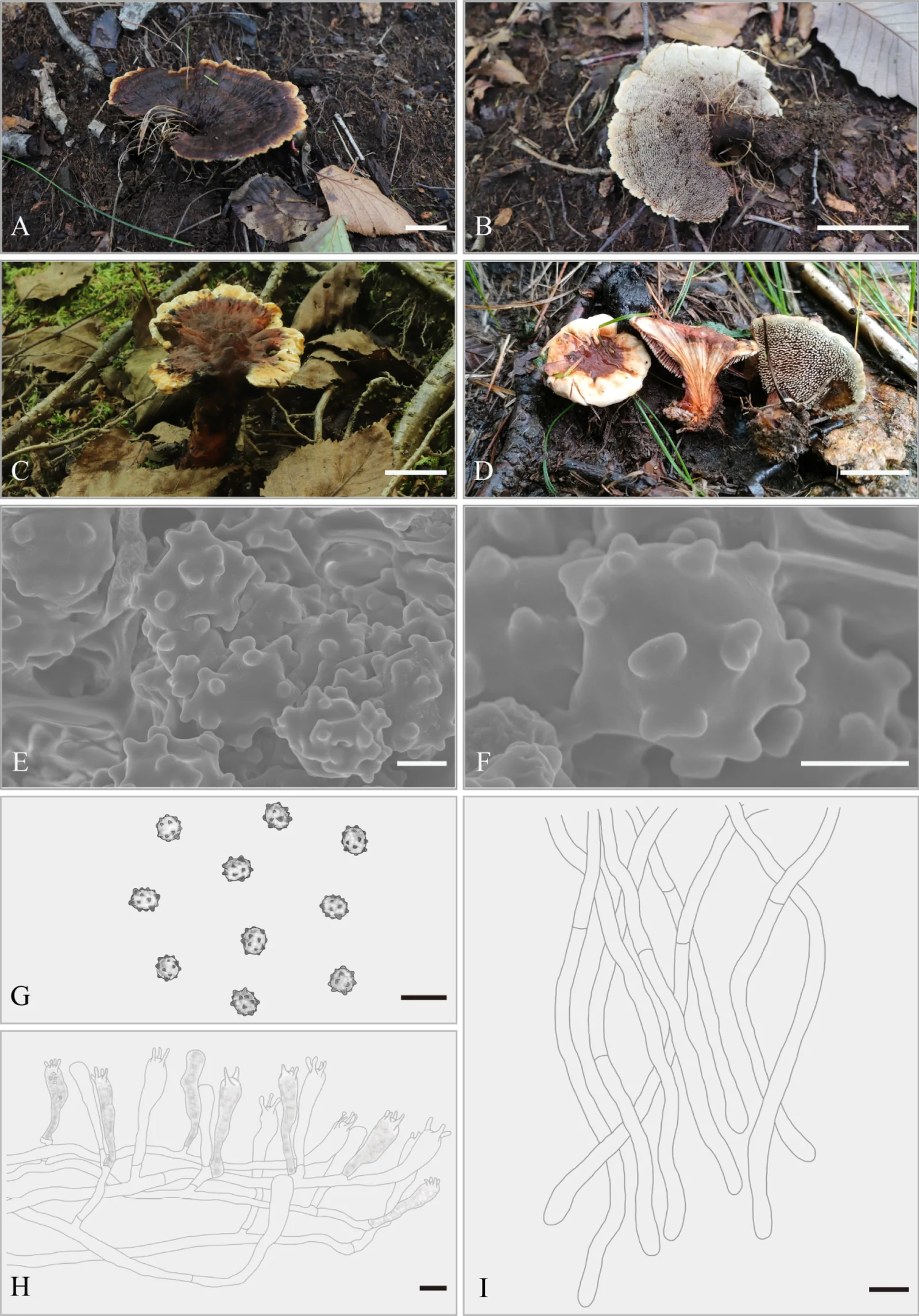
*Hydnellum flavomarginatum*. **A–D**. Basidiocarps; **E, F**. SEM of basidiospores (SEM images from FJAU81639); **G–I**. Microscopic structures (drawn from FJAU81639); **G**. Basidiospores; **H**. Hymenium and subhymenium; **I**. Pileipellis. Scale bars: 2 cm (**A–D**); 2.5 μm (**E, F**); 5 μm (**G–I**).

##### Description.

Basidiocarps small to medium, solitary to gregarious, 42.2–78.1 mm in height, and leathery when fresh. Pileus 32.5–100.6 mm broad when mature, brownish orange (6C8) to dark brown (6F8), circular to subcircular, center depressed; pileus margin has golden yellow (5B7) tomentum and displays concentric zones, with the coloration darkening towards the center. Pileus context 5.5–11.2 mm thick, woody, yellow white (4A2) to deep orange (6A6). Spines sub-decurrent, white (7A1) to greyish brown (6F3), conical, slightly sparse, 1–2 spines/mm^2^, 0.5–2.5 mm long, 0.1–0.5 mm in diameter. Stipe 25.3–50.2 × 8.0–15.5 mm, covered with dark brown (6F2) tomentum, leathery when fresh, central to slightly eccentric, cylindrical and somewhat inflated at the base, and solid. Odor mild, resembling chocolate.

Basidiospores 6.0–7.0 × 6.0–6.5 (7.0) μm, av. L = 6.65 μm, av. W = 6.19 μm, Q = 1.00–1.16, av. Q = 1.07, globose to subglobose, brown (6E5), thin-walled, tuberculate, MR+, LCB+; tuberculi usually isolated or grouped in two, 0.3–0.5 μm long, 0.3–1.0 μm in diameter. Basidia 30.5–42.5 (48.5) × 7.5–9.0 μm, clavate to suburniform, some with granular contents; sterigmata 2–4, 3.5–4.0 × 0.5–0.8 µm, conical. Basidioles 25.5–30.0 × 6.0–8.0 μm, smaller than basidia. Subhymenium trama filamentous, hyphae 3.5–4.0 μm wide, septate, thin-walled, hyaline in 5% KOH. Hyphae of pileus context 4.5–5.0 μm, thin-walled. Pileipellis composed of cylindrical hyphae, slightly interwoven, rarely branched, terminal elements cylindrical at apex, cells 55.5–175.5 × 4.5–5.0 μm, clamp connections occasionally observed. Hyphae from the inner layer of stipe, 3.0–4.0 μm wide, subparallel, occasionally branched. Hyphae from the surface layer of stipe, 4.0–4.5 μm wide, interwoven, occasionally branched.

##### Habitat and distribution.

The species occurs gregariously in a *Q. mongolica* forest within the core zone of the Ji’an National Nature Reserve, Ji’an City, China. The site is located at an elevation of 600–700 m, characterized by a north temperate continental climate and minimal human disturbance. Specimens were collected adjacent to three separate *Q. mongolica* trees at distances of 212.3 cm (DBH = 20.8 cm), 50.4 cm (DBH = 20.7 cm), and 115.6 cm (DBH = 27.5 cm). At the time of collection, the environmental parameters for the three specimens were as follows: soil temperature was 15.9 °C, 16.2 °C, and 15.5 °C; soil volumetric water content was 20.2%, 26.4%, and 19.6%; soil pH was 5.2, 4.9, and 5.3; air temperature was 16.9 °C, 17.2 °C, and 16.8 °C; relative humidity was 90.2%, 90.4%, and 89.5%.

##### Additional specimens examined.

China • Jilin Province, Ji’an City, Ji’an National Nature Reserve, 41°16'18"N, 126°7'10"E, Elevation: ca. 685 m; on soil in *Q. mongolica* forest, 11 Sep 2020, Yonglan Tuo FJAU81641; GenBank numbers: ITS = PX626419, nrLSU = PX623210. China • Ji’an City, Ji’an National Nature Reserve, 41°16'9"N, 126°7'44"E, Elevation: ca. 606 m; on soil in *Q. mongolica* forest, 11 Sep 2020, Yonglan Tuo FJAU81640; GenBank numbers: ITS = PX626416, nrLSU = PX623209.

##### Notes.

Macromorphologically, *H. flavomarginatum* is similar to *H. aurantiacum* when young, featuring a depressed pileus with a brownish orange (6C8) center and a white (4A1) tomentose margin. Upon maturity, however, *H. flavomarginatum* develops a uniformly dark brown (6F8), concentrically zonate pileus, whereas *H. aurantiacum* remains orange and azonate, developing a pitted, scrobiculate, and radially rugose surface that bruises black. The spines of *H. flavomarginatum* are sub-decurrent, sparse (1–2 spines/mm^2^, 0.5–2.5 mm long), and white to greyish brown, contrasting with the fully decurrent, crowded, longer spines (up to 4 mm) of *H. aurantiacum*. Finally, the two species differ in odor: *H. aurantiacum* is unpleasant, whereas *H. flavomarginatum* possesses a mild, chocolate-like odor.

Micromorphologically, the basidiospores of *H. flavomarginatum* are globose to subglobose, 6.0–7.0 × 6.0–6.5 μm, ornamented with isolated or paired tubercles. In contrast, those of *H. aurantiacum* are larger (5.5–8(–9.3) × 5.5–6.5(–7) μm) and bear prominent, flattened to exsculptate tubercles. A critical distinction lies in the presence of clamp connections: *H. flavomarginatum* possesses them occasionally in the pileipellis, whereas *H. aurantiacum* lacks them in all tissues examined (pileus, stipe, and spine). Additionally, *H. aurantiacum* contains gloeopleurous-like hyphae up to 7 μm in diameter in both the pileus and stipe—such specialized hyphae have not been observed in *H. flavomarginatum*.

Ecologically, *H. flavomarginatum* is currently known only from northeastern China (Jilin Province), where it occurs solitary to gregarious in montane *Q. mongolica* forests at 600–700 m elevation. In contrast, *H. aurantiacum* is widespread and common in hardwood forests throughout north and central Florida.

Phylogenetic analyses based on concatenated ITS and nrLSU sequences revealed that *H. flavomarginatum* forms a distinct, well-supported clade (MLBS = 100%, BPP = 1.0), sister to *H. aurantiacum*, further corroborating their genetic divergence.

#### Hydnellum imbricatum

Taxon classificationFungiThelephoralesBankeraceae

Yonglan Tuo, Bo Zhang & Yu Li sp. nov.

31DDE56B-B533-5665-B378-2D07711ACE00

Fungal Names: FN 573073

Fig. 6A–I

##### Etymology.

The specific epithet “*imbricatum*” refers to the layered, imbricate scales on the pileus.

##### Holotype.

China • Jilin Province, Ji’an City, Ji’an National Nature Reserve, 41°16'25"N, 126°7'18"E, Elevation: ca. 782 m, on soil in a *Pinus koraiensis* Siebold & Zucc. forest, 11 September 2020, Yonglan Tuo FJAU81633. GenBank numbers: ITS = PX626422, nrLSU = PX623214.

##### Diagnosis.

Basidiocarps medium to large, solitary, with a distinct chocolate odor. Pileus 62.5–140.5 mm wide, brownish yellow to dark brown, with concentric scales (large and fissured at center, diminishing toward margin); margin incurved, tomentose. Spines decurrent, white to yellowish white, sparse (1–2/mm^2^). Stipe short (25.0–32.0 mm), shortly spinose at apex, white tomentum. Spores relatively large, ellipsoid, av. 6.18 × 5.13 μm, brown, with tubercles solitary or clustered.

**Figure 6.**
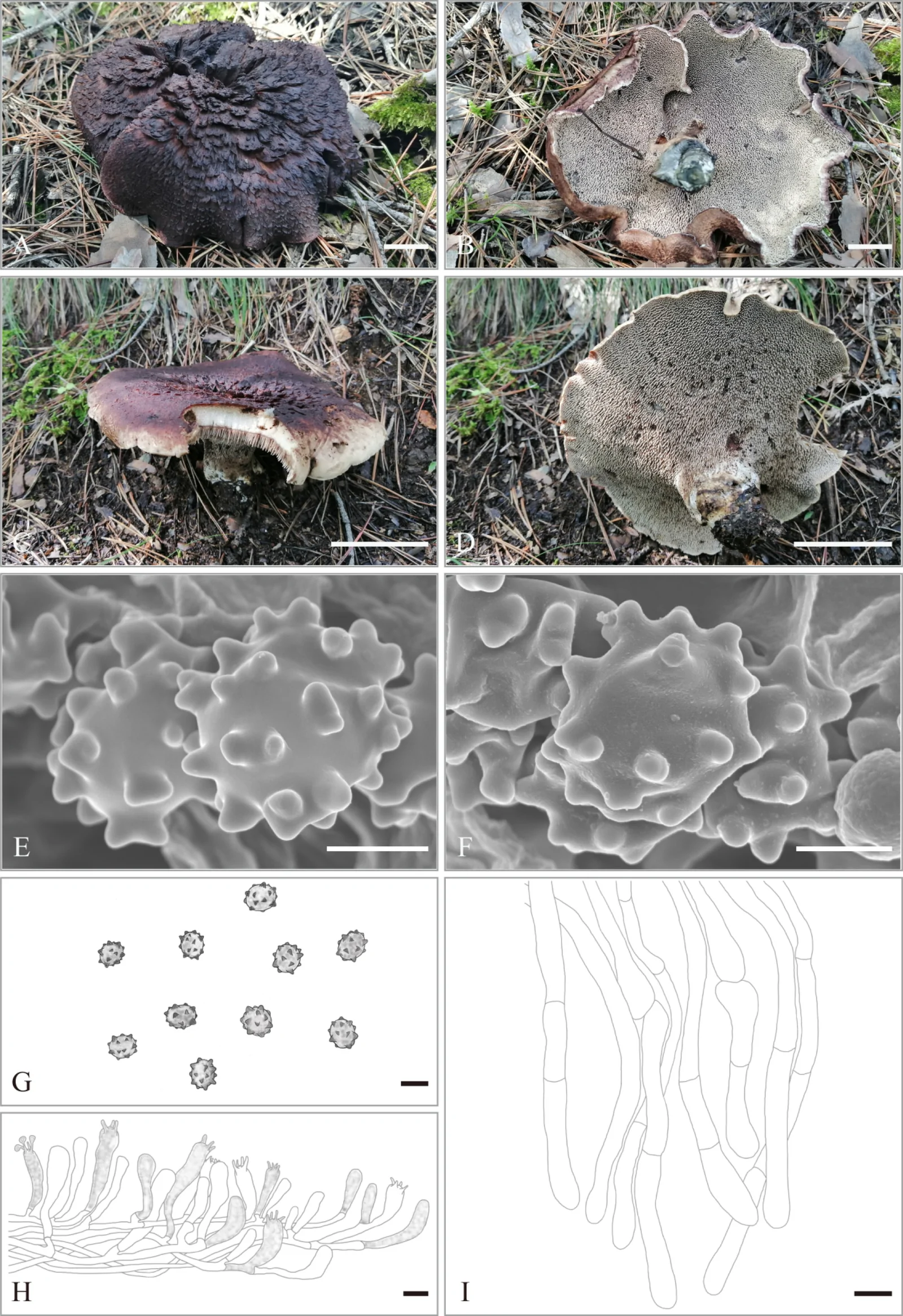
*Hydnellum imbricatum*. **A–D**. Basidiocarps; **E, F**. SEM of basidiospores (SEM images from FJAU81633); **G–I**. Microscopic structures (drawn from FJAU81633); **G**. Basidiospores; **H**. Hymenium and subhymenium; **I**. Pileipellis. Scale bars: 2 cm (**A–D**); 2.5 μm (**E, F**); 5 μm (**G)**; 10 μm (**H, I)**.

##### Description.

Basidiocarps medium to large, solitary, fleshy when fresh, 40.0–70.0 mm in height. Pileus 62.5–140.5 mm wide, brownish yellow (5C7) to dark brown (7F7), azonate, round to irregular, plano-convex or depressed in the center; cracked by fissures forming large scales in the center, becoming depressed and small scales toward margin, scales arranged somewhat concentrically, brownish yellow (5C7) to dark brown (7F7), discoloration not observed; margin incurved, covered with white (4A1) tomentum. Pileus context white (4A1), 10.0–15 mm thick. Spines decurrent, white to yellowish white (4A1–4A2), slightly sparse, 1–2 spines/mm^2^, 4.8–8.5 mm long, 0.5–1.0 mm in diameter. Stipe 25.0–32.0 × 7.0–23.0 mm, cylindrical to attenuate below, covered by short spines in the upper stipe, solid, central to slightly eccentric, surface covered with white (4A1) tomentum. Odor mild, resembling chocolate.

Basidiospores (5.0) 6.0–6.5 (7.0) × (4.5) 5.0–5.5 (6.0) μm, av. L = 6.18 μm, av. W = 5.13 μm, Q = (1.09) 1.12–1.16 (1.40), av. Q = 1.20, subglobose to ellipsoid, brown, thin-walled, tuberculate, MR–, LCB+; tuberculi usually isolated or grouped in 2 or more, 0.4–0.9 μm long, 0.3–1.4 μm in diameter. Basidia (25.5) 30.0–38.0 (45.0) × (6.0) 6.5–7.0 (8.0) μm, clavate to suburniform, some with granular contents; sterigmata 2–4, 4.0–5.0 × 0.5–1.0 μm, conical. Basidioles 20.0–25.0 × (5.5) 6.0–7.0 (7.5) μm, smaller than basidia, some with granular contents. Subhymenium trama filamentous, hyphae 3.0–4.0 μm wide, septate, thin-walled, hyaline in 5% KOH. Hyphae of pileus context 8.5–10.0 μm, thin-walled. The pileipellis is composed of cylindrical hyphae, slightly interwoven, rarely branched, terminal elements cylindrical at apex, cells 20.0–70.0 × 6.0–8.0 μm. Hyphae from the inner layer of stipe, 6.5–7.5 μm wide, subparallel, occasionally branched. Hyphae from the surface layer of stipe, 5.0–6.5 μm wide, slightly interwoven, occasionally branched. All the hyphae MR–, LCB–; clamp connections not observed.

##### Habitat and distribution.

The species was found growing solitarily in a forest of *Pinus koraiensis* within the core zone of the Ji’an National Nature Reserve, Ji’an City, China (ca. 600–800 m; North Temperate Continental Climate). The site experiences minimal human disturbance. Two specimens were collected at distances of 137.5 cm (DBH = 25.24 cm) and 78.5 cm (DBH = 15.5 cm) from *P. koraiensis* trees, respectively. At the time of collection, the environmental parameters for the two specimens were as follows: soil temperature was 17.5 °C and 17.9 °C; soil volumetric water content was 21.6% and 18.3%; soil pH was 6.1 and 6.2; air temperature was 19.7 °C and 19.5 °C; relative humidity was 79.2% and 74.8%.

##### Additional specimens examined.

China • Jilin Province, Ji’an City, Ji’an National Nature Reserve, 41°16'12"N, 126°7'47"E, Elevation: ca. 632 m, on soil in a *P. koraiensis* forest, 11 September 2020, Yonglan Tuo FJAU81634. GenBank numbers: ITS = PX626423, nrLSU = PX623215.

##### Notes.

*Hydnellum imbricatum* is morphologically similar to *H. lidongensis*, sharing a brown, scaly pileus with an incurved, plano-convex to slightly depressed margin, a monomitic hyphal system lacking clamp connections, and ellipsoid, brown, thin-walled, tuberculate basidiospores with isolated or small-clustered tuberculi. However, the two species differ significantly in their macromorphology, micromorphology, and ecology.

Macromorphologically, *H. imbricatum* is distinguished by its much larger basidiocarps (pileus 62.5–140.5 mm vs. up to 35 mm across). The pileus is uniformly brown with large, concentric, radiating scales and a white-tomentose margin (*H. lidongensis*: purplish-brown at the margin, dark brown at the center when fresh, with smaller, irregularly arranged, rimose scales and non-tomentose margin). Its spines are conspicuously longer (4.8–8.5 mm vs. up to 1 mm) and sparser (1–2 vs. 4–6 spines​ per mm^2^). The stipe is shorter (25–32 × 7–23 mm vs. up to 45 × 10 mm), white to grayish-white, covered with white tomentum, and bearing short spines on the upper portion (*H. lidongensis*: longer, slender, brown, fibrillose, and lacking both a​ tomentum and spines). The two species also differ markedly in odor: *H. imbricatum* has a chocolate odor whether fresh or dried, whereas dried *H. lidongensis* acquires​ a farinaceous odor and a bitter taste.

Micromorphologically, *H. imbricatum* differs from *H. lidongensis* in having slightly larger basidiospores (av. L × W = 6.18 × 5.13 μm vs. 5.5 × 4.9 μm; av. Q = 1.12–1.16 vs. 1.15–1.20), longer sterigmata (4.0–5.0 μm vs. 2.0–3.0 μm), and generally longer basidia (30.0–38.0 μm vs. 19.2–39.3 μm).

Ecologically, *H. imbricatum* is solitary in temperate *P. koraiensis* forests at mid elevations (ca. 600–800 m) in Jilin Province, northeastern China, in temperate continental climate with slightly acidic soils (pH 6.1–6.2). In contrast, *H. lidongensis* is solitary in subtropical montane *Fagaceae* forests at higher elevations (ca. 2400 m) in Yunnan Province, southwestern China.

Phylogenetically, the combined ITS + nrLSU analysis (Fig. 1) reveals that *H. imbricatum* forms a strongly supported monophyletic lineage (MLBS = 100%, BPP = 1.00) sister to *H. lidongensis*. Supported by both morphological and molecular evidence, the two species are unequivocally distinct.

#### Hydnellum jianense

Taxon classificationFungiThelephoralesBankeraceae

Yonglan Tuo, Bo Zhang & Yu Li sp. nov.

F75331D8-D9BB-5F5C-B618-ABF3A6E176AF

Fungal Names: FN 573077

Fig. 7 A–G

##### Etymology.

The specific epithet “*jianense*” is derived from Ji’an City, Jilin Province, China, which is the type locality of this species.

##### Holotype.

China • Jilin Province, Ji’an City, Ji’an National Nature Reserve, 41°16'21"N, 126°7'16"E, Elevation: ca. 728 m, on soil in a *Quercus mongolica* Fisch. ex Ledeb. forest, 16 September 2021, Yonglan Tuo FJAU81642. GenBank numbers: ITS = PX626417, nrLSU = PX623216.

##### Diagnosis.

Basidiocarps small, solitary, leathery when fresh. Pileus 22.0–30.0 mm wide, white to yellowish grey, plano-convex, covered with grayish orange tomentum and bruising light brown; context dark brown, woody, distinctly stratified. Spines subdecurrent, white to dark brown, sparse (1–2/mm^2^). Stipe short (15.0–20.2 × 10.2–15.0 mm), with light brown tomentum above and white tomentum at base. Basidiospores small, ellipsoid, av. 4.98 μm × 4.14 μm, brown, thin-walled, with isolated amyloid tubercles, amyloid; pileipellis terminal cells exceptionally long (105.5–170.0 μm). Associated with *Q. mongolica*.

**Figure 7.**
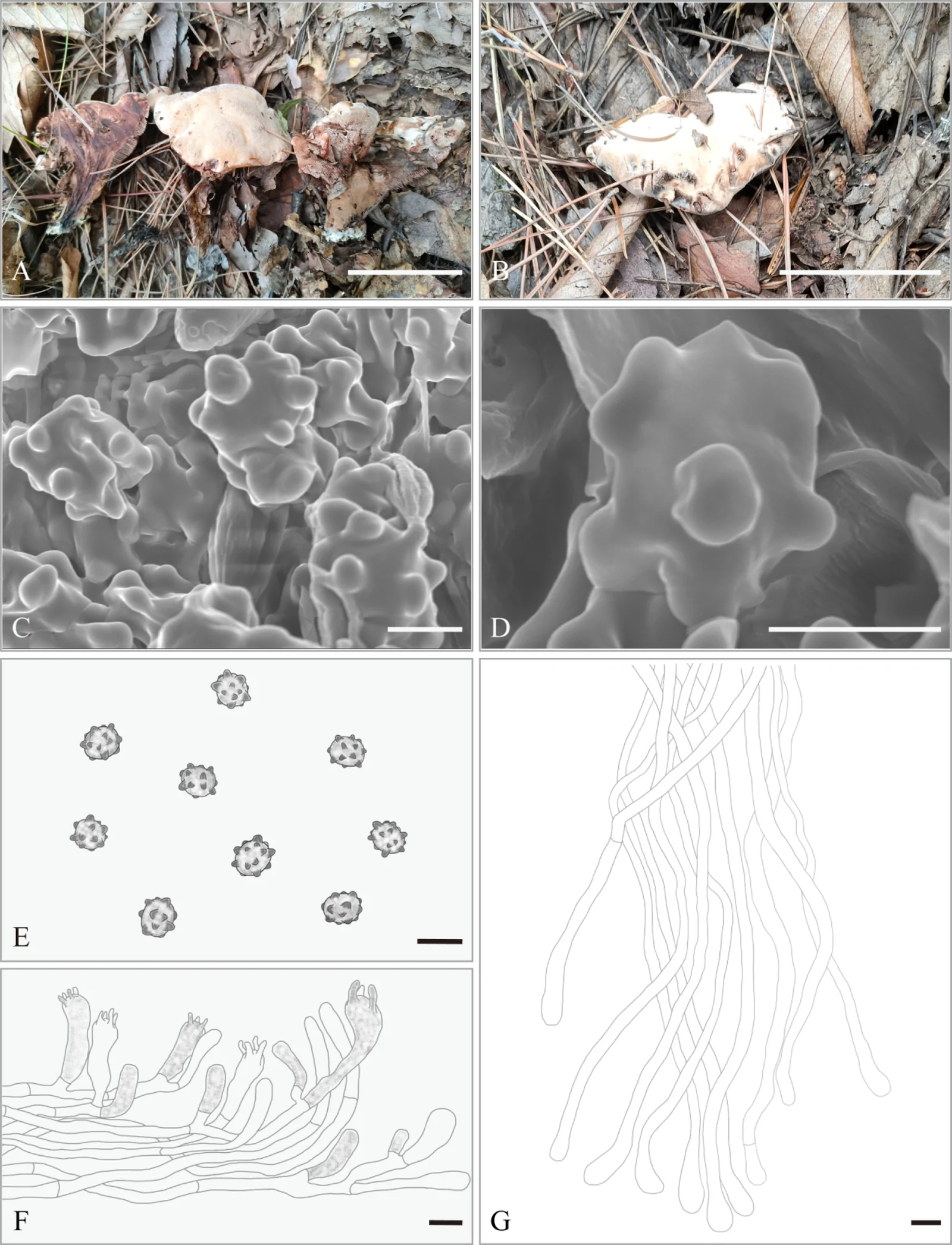
*Hydnellum jianense*. **A, B**. Basidiocarps; **C, D**. SEM of basidiospores (SEM images from FJAU81642); **E–G**. Microscopic structures (drawn from FJAU81642); **E**. Basidiospores; **F**. Hymenium and subhymenium; **G**. Pileipellis. Scale bars: 2 cm (**A, B**); 2.5 μm (**C, D**); 5 μm (**E–G)**.

##### Description.

Basidiocarps small to medium, solitary, 55.2–65.4 mm in height, leathery when fresh. Pileus 22.0–30.0 mm broad, white (6A1) to yellowish grey (4B2), subcircular, plano-convex, covered with greyish orange (6B3) tomentum and becomes light brown (6D4) when touched. Pileus context 7.5–10.5 mm thick, dark brown (6F5), woody, and distinctly layered. Spines sub-decurrent, conical, white (7A1) to dark brown (7F8), slightly sparse, 1.0–3.0 mm long and 0.2–0.5 mm in diameter, at a density of 1–2 spines/mm^2^. Stipe 15.0–20.2 × 10.2–15.0 mm, central to slightly eccentric, cylindrical to attenuate below, solid; covered with light brown (6D5) tomentum on the upper part and white (6A1) tomentum at the base. Odor mild.

Basidiospores 4.5–5.0 × 4.0–4.5 (5.0) μm, av. L = 4.98 μm, av. W = 4.14 μm, Q = 1.11–1.25, av. Q = 1.20, ellipsoid, brown (6E5), thin-walled, tuberculate, MR+, LCB+; tuberculi usually isolated, 0.5–0.8 μm long, 0.6–1.5 μm in diameter. Basidia 25.5–33.5 × 6.0–7.0 μm, clavate to suburniform, some with granular contents; sterigmata 2.5–3.0 × 0.25–0.5 μm, conical. Basidioles 20.0–25.0 × 5.0–6.0 μm, smaller than basidia. Subhymenium trama filamentous, hyphae 3.0–4.0 μm wide, septate, thin-walled, hyaline in 5% KOH. Hyphae of pileus context 4.0–5.0 μm, thin-walled. Pileipellis composed of cylindrical hyphae, slightly interwoven, terminal elements cylindrical at apex, cells 105.5–170.0 × 5.0 μm. Hyphae from the inner layer of stipe, 5.0–6.0 μm wide, subparallel. Hyphae from the surface layer of stipe, 5.0 μm wide, interwoven, occasionally branched.

##### Habitat and distribution.

The species occurs gregariously in a *Q. mongolica* forest within the core zone of the Ji’an National Nature Reserve, Ji’an City, China (ca. 700–800 m, north temperate continental climate), an area with minimal human disturbance. Two specimens were collected at distances of 242.5 cm (DBH = 10.8 cm) and 115.6 cm (DBH = 15.4 cm) from *Q. mongolica* trees, respectively. At the time of collection, the environmental parameters for the two specimens were as follows: soil temperature was 15.8 °C and 15.9 °C; soil volumetric water content was 19.1% and 20.8%; soil pH was 5.8 and 5.6; air temperature was 16.5 °C and 16.7 °C; and relative humidity was 94.7% and 91.5%, respectively.

##### Additional specimens examined.

China • Jilin Province, Ji’an City, Ji’an National Nature Reserve, 41°16'19"N, 126°7'16"E, Elevation: ca. 715 m, on soil in a *Q. mongolica* forest, 16 September 2021, Yonglan Tuo FJAU81643. GenBank numbers: ITS = PX626418, nrLSU = PX623217.

##### Notes.

Morphologically similar to *H. ferrugineum*, *H. jianense* is characterized by solitary basidiocarps with grayish-orange (6B3) tomentum that darkens when bruised, a monomitic hyphal system with simple-septate generative hyphae, and brown, tuberculate basidiospores. However, the two species differ markedly in their macromorphological, micromorphological, and ecological characters.

Macromorphologically, *H. jianense* is distinguished from *H. ferrugineum* by its smaller basidiocarps (pileus 22.0–30.0 mm vs. 30–100 mm); azonate, white to yellowish-grey pileus (vs. zonate, aging from whitish to reddish brown); grayish-orange tomentum that discolors to light brown when bruised but never exudes reddish droplets (vs. exuding such droplets when immature); and yellowish-brown, distinctly layered context, dark brown when cut (vs. reddish-brown and not distinctly layered).

Micromorphologically, *H. jianense* is distinguished from *H. ferrugineum* by its smaller, ellipsoid basidiospores (4.5–5.0 × 4.0–4.5 μm vs. 5.8–6.3 × 3.6–4.5 μm), amyloid (vs. inamyloid), and longer basidia (25.5–33.5 × 6.0–7.0 μm vs. 20–30 × 5–6 μm). Additionally, whereas oleiferous hyphae are present in *H. ferrugineum*, they are absent in *H. jianense*.

Ecologically, *H. jianense* occurs gregariously in temperate broad-leaved forests dominated by *Q. mongolica* at 700–800 m elevation in Jilin Province, northeastern China. The region is characterized by a north temperate continental climate, acidic soils (pH 5.6–5.8), and high relative humidity (91.5–94.7%). In contrast, *H. ferrugineum* is typically associated with *Pinus* spp. in coniferous forests and widely distributed across Europe.

Phylogenetically, the combined ITS + nrLSU analysis (Fig. 2) reveals that *H. jianense* constitutes a strongly supported monophyletic lineage (MLBS = 100%, BPP = 1.00) sister to *H. ferrugineum* (MLBS = 100%, BPP = 1.00). The congruence of morphological, ecological, and phylogenetic data supports the recognition of *H. jianense* as a distinct species.

### Key to species of *Hydnellum* in China

**Table:** 

1	Pileus depressed to infundibuliform	**2**
–	Pileus irregularly to circular	**33**
2	Pileus zonate	**3**
–	Pileus azonate	**17**
3	Pileus surface smooth	**4**
–	Pileus surface covered tomentum	**7**
4	Pileus > 70 mm wide, spines grayish brown	***H. rubidofuscum***
–	Pileus < 70 mm wide, spines grayish blue to brown	**5**
5	Pileus reddish brown, spines grayish blue	***H. qinghaiense***
–	Pileus light brown to dark brown	**6**
6	Basidiospores < 4.0 μm wide on average, spines reddish brown	***H. concentricum***
–	Basidiospores > 4.0 μm wide on average, spines brown	***H. sulcatum***
7	Pileus covered with white tomentum	**8**
–	Pileus tomentum yellow to dark brown	**11**
8	Basidiospores > 6.0 μm long on average, spines white	***H. aurantiacum***
–	Basidiospores < 6.0 μm long on average	**9**
9	Basidiospores 5.0–6.0 μm long, pileus tomentum white	***H. fuscozonatum***
–	Basidiospores ≤ 5.0 μm long	**10**
10	Pileus brownish orange, zonate grayish yellow	***H. aureomarginatum***
–	Pileus and zonate white to light orange	***H. peckii***
11	Basidiospores > 6.5 μm wide on average, pileus tomentum golden yellow	***H. flavomarginatum***
–	Basidiospores < 6.5 μm wide on average, pileus tomentum pale orange to light brown	**12**
12	Basidiospores 5.0–6.0 μm long	**13**
–	Basidiospores 3.0–4.8 μm long	**14**
13	Basidiospores ≥ 6.0 μm long on average, spines brownish grey	***H. ferrugineum***
–	Basidiospores < 6.0 μm long on average, spines infundibuliform	***H. atrorubrum***
14	Pileus ≥ 50 mm wide, pileus center depression	**15**
–	Pileus < 50 mm wide, pileus infundibuliform to subinfundibuliform	**16**
15	Pileus brownish yellow to brown, spines brownish yellow	***H. subalpinum***
–	Pileus light orange to mars orange, spines light orange	***H. chrysinum***
16	Pileus irregularly circular, zonate brown	***H. bomiense***
–	Pileus flabelliform, zonate grayish red to dark brown	***H. yunnanense***
17	Pileus smooth	**18**
–	Pileus covered tomentum	**22**
18	Spines whitish, basidiospores > 5.5 μm long on average	**19**
–	Spines brown, basidiospores < 5.5 μm long on average	**20**
19	Pileus ochraceous to fulvous brown, habitat in *Quercus* forest	***H. illudens***
–	Pileus ochraceous brownish yellow to reddish brown habitat in *Pinus* and *Abies* forest	***H. subscabrosellum***
20	Basidiospores > 5.0 μm long on average, pileus black brown	***H. fagiscabrosum***
–	Basidiospores < 5.0 μm long on average	**21**
21	Pileus 70–90 mm wide, light orange to deep orange	***H. earlianum***
–	Pileus < 60 mm wide, light brown to brown	***H. succulentus***
22	Pileus tomentum white, spines carrot red to bruising black	***H. complicatum***
–	Pileus tomentum pale yellow to dark brown	**23**
23	Pileus tomentum yellowish to brownish yellow	**24**
–	Pileus tomentum brownish orange to dark brown	**29**
24	Spines golden yellow or yellowish brown	**25**
–	Spines whitish	**26**
25	Spines 0.5–1.5 mm long, golden yellow	***H. aureoluteum***
–	Spines 1.0–6.7 mm, yellowish brown	***H. pileospinosum***
26	Basidiospores < 5.0 μm long on average, pileus > 150 mm wide	***H. versipelle***
–	Basidiospores > 5.0 μm long on average, pileus < 150 mm wide	**27**
27	Basidiospores 5.0–6.0 μm long on average, pileus light brown	***H. martioflavum***
–	Basidiospores 6.0–6.5 μm long on average, pileus dark brown to reddish brown	**28**
28	Pileus dark brown, habitat in *P. koraiensis* forest	***H. imbricatum***
–	Spines reddish brown, habitat in *Pinus* and *Quercus* mixed forest	***H. edule***
29	Spines white to pale yellow, pileus pale orange	***H. grosselepidotum***
–	Spines pale orange to dark brown	**30**
30	Basidiospores < 5.0 μm long on average, spines golden yellow	***H. brunneorubrum***
–	Basidiospores > 5.0 μm long on average, spines orange to brown	**31**
31	Basidiospores > 6.0 μm long on average, pileus tomentum black	***H. ailaoense***
–	Basidiospores < 6.0 μm long on average, pileus tomentum orange to brown	**32**
32	Pileus tomentum, spines pale orange to dark brown	***H. spongiosipes***
–	Pileus tomentum light brown, spines grayish orange to brown	***H. lidongense***
33	Pileus pastel red to dark brown, zonate	**34**
–	Pileus pastel yellow to black, azonate	**42**
34	Pileus smooth	**35**
–	Pileus covered tomentum	**37**
35	Pileus 10–50 mm wide, spines and pileus zonate reddish brown	***H. tarda***
–	Pileus 60–120 mm wide, spines reddish brown to dark violet	**36**
36	Spines dark violet, basidiospores < 5.0 μm long on average	***H. atrospinosum***
–	Spines dark brown, basidiospores < 5.0 μm long on average	***H. nitidum***
37	Pileus tomentum light brown to dark brown	**38**
–	Pileus tomentum others color	**40**
38	Spines < 2.0 μm long on average, pinkish white	***H. fibulatum***
–	Spines > 2.0 μm long on average, olivaceous buff to dark brown	**39**
39	Pileus zonate dark blue, spines light brown to dark brown	***H. suaveolens***
–	Reddish brown to dark brown, spines olivaceous buff	***H. subcaeruleum***
40	Pileus tomentum pastel red to dark magenta, pileus pastel red	***H. squamulosum***
–	Pileus tomentum yellowish to orange brown	**41**
41	Basidiospores > 5.0 μm long on average, zonate dark yellowish	***H. cinnamomeum***
–	Basidiospores < 5.0 μm long on average, zonate orange brown	***H. xanthopus***
42	Pileus smooth	**43**
–	Pileus covered tomentum	**47**
43	Basidiospores ≥ 5.0 μm long on average, pileus grayish brown to black	**44**
–	Basidiospores < 5.0 μm long on average, pileus light yellow to grayish brown	**45**
44	Basidiospores > 5.0 μm wide on average, pileus greyish brown	***H. subailaoense***
–	Basidiospores < 5.0 μm wide on average, pileus black	***H. melanocarpum***
45	Basidiospores ≥ 4.0 μm wide on average, spines white to dark brown	**46**
–	Basidiospores < 4.0 μm wide on average, spines black	***H. radiatum***
46	Pileus irregularly circular, spines white	***H. inflatum***
–	Pileus irregularly flabelliform, spines dark brown	***H. granulosum***
47	Pileus tomentum white to light brown	**48**
–	Pileus tomentum orange gray to dark brown	**50**
48	Basidiospores < 5.0 μm wide on average, pileus chocolate	***H. chocolatum***
–	Basidiospores ≥ 5.0 μm wide on average, pileus orange to brownish yellow	**49**
49	Pileus 8.0–15.0 mm wide, spines brownish yellow	***H. jianense***
–	Pileus 63.6–80.5 mm wide, spines white to yellowish white	***H. coalescens***
50	Spines yellowish white to orange white	**51**
–	Spines orange white to dark brown	**52**
51	Basidiospores > 5.0 μm wide on average, pileus tomentum reddish brown to dark brown	***H. coactum***
–	Basidiospores < 5.0 μm wide on average, pileus tomentum yellow to dark blonde	***H. caeruleum***
52	Pileus tomentum orange gray, brown upon contac	***H. aureotomentosum***
–	Pileus tomentum reddish brown to grayish brown	***H. crassipileatum***

## Discussion

Through an integrated analysis of morphological and molecular phylogenetic data, this study describes four new *Hydnellum* species from Jilin Province and one from Anhui Province, China, thereby enriching our understanding of the genus’s diversity in Northeast and Central China. Furthermore, we summarize the key morphological and ecological characteristics of documented *Hydnellum* species in China (Suppl. material 1) to facilitate accurate identification and to advance the understanding of their ecological habits.

Typically, microscopic features (e.g., spore and basidia sizes) are effective for distinguishing closely related species (Banker 1906; Harrison 1964; Hrouda 1999; Nitare et al. 2021). Based on these characteristics, the four new species described in this study can be clearly distinguished from similar species: *H. imbricatum* vs. *H. lidongense* (average L × W = 6.18 × 5.13 μm vs. 5.5 × 4.9 μm); *H. coalescens* vs. *H. edulium* (spore length = 5.0–6.0 μm vs. 4.5–5.5 μm); *H. flavomarginatum* vs. *H. aurantiacum* (basidiospore width = 6.0–6.5 μm vs. 4.3–4.9 μm); *H. jianense* vs. *H. ferrugineum* (basidiospore size = 4.5–5.0 × 4.0–4.5 μm vs. 5.6–6.4 × 4.8–5.2 μm). However, overlap of morphological features exists among other documented species. For instance, *H. aureomarginatum* and *H. earlianum* are highly similar morphologically, with both species exhibiting orange pilei and nearly identical spore sizes (5.0–6.0 × 4.0–5.0 μm vs. 5.0–5.5 × 4.0–5.0 μm). Consequently, accurate identification of many *Hydnellum* species based solely on a single morphological characteristic is difficult or even impossible, which has constrained taxonomic research within this genus.

In contrast, an integrated approach combining morphological characteristics, molecular sequence analysis, host information, and other relevant data has demonstrated high effectiveness in distinguishing species within the genus *Hydnellum* (Parfitt et al. 2007; Larsson et al. 2019; Wang et al. 2024; Song et al. 2025). *H. aureomarginatum* has a pileus margin with distinct annular zones and a significantly smaller pileus diameter (1.76–4.23 cm vs. 7.0–9.0 cm). Their habitats also differ substantially: *H. earlianum* inhabits mixed forests of *Tsuga candelensis* and Rhododendron, whereas *H. aureomarginatum* occurs in *Q. mongolica* forests. Furthermore, in the phylogenetic tree, the sequences of *H. aureomarginatum* form a distinct and well-supported lineage (BS = 100%, BPP = 1.00), sister to *H. earlianum* (BS = 100%, BPP = 0.95). This indicates that an approach combining phylogenetic and morphological analyses is effective in objectively elucidating the diversity and phylogenetic relationships of *Hydnellum* species (Baird et al. 2013; Larsson et al. 2019; Mu et al. 2021; Song et al. 2022; Song et al. 2025).

The habitat preference of *Hydnellum* species appears to be strongly associated with forests dominated by *Pinaceae*, *Fagaceae*, or mixed stands (Agerer 1993; Arnolds 2010; Nitare et al. 2021). Our collection data support this: *H. coalescens* was found in a *Q. serrata* forest; *H. aureomarginatum*, *H. flavomarginatum*, and *H. jianense* were collected in *Q. mongolica* forests; while *H. imbricatum* was recorded in a *P. koraiensis* forest. These findings suggest potential ecological associations between the five species and specific plant groups, namely *Fagaceae* (particularly the genus *Quercus*) and *Pinaceae*. Furthermore, an analysis of the ecological records for the 48 documented species of *Hydnellum* in China reveals that 40 are found primarily in forests of *Quercus* or *Pinaceae* (Suppl. material 1). This indicates a strong association with these forest types, a factor that can aid in the discovery and identification of species within this genus. However, the existing records for these species lack detailed data on host specificity and fine-scale ecological habits. We therefore recommend that future fieldwork prioritize collecting detailed ecological metadata during specimen collection, including specific host tree identity, distance to the host, host age, soil moisture, and soil pH. The integration of such newly collected environmental data with existing morphological and ecological evidence will not only enable a more robust species identification framework but also facilitate the prediction and discovery of novel *Hydnellum* species and their distributions, thereby informing targeted conservation efforts.

## Supplementary Material

XML Treatment for Hydnellum aureomarginatum

XML Treatment for Hydnellum coalescens

XML Treatment for Hydnellum flavomarginatum

XML Treatment for Hydnellum imbricatum

XML Treatment for Hydnellum jianense

## Additional information

### Artificial Intelligence (AI) use

The authors accept full responsibility for the content of the manuscript, including the disclosure of any use of AI.

No AI tools were used in the preparation of this manuscript.

### Author ORCIDs

Y. Tuo https://orcid.org/0000-0001-6019-1038

Z. Qi https://orcid.org/0000-0002-0037-9407

J. Hu https://orcid.org/0000-0002-7562-7612

B. Zhang https://orcid.org/0000-0001-9508-8188

Y. Li https://orcid.org/0000-0003-4719-7210

### Data availability

ML tree, BI tree, and alignment files have been uploaded to the online database figshare (https://doi.org/10.6084/m9.figshare.32038755.V2). The comparison of morphological characteristics of Chinese *Hydnellum* species has been provided in the Suppl. material 1.

## Supplementary materials

Supplementary material 1 Morphological comparison of *Hydnellum* species in China Data type xlsx https://binary.pensoft.net/file/1811085This dataset is made available under the Open Database License (http://opendatacommons.org/licenses/odbl/1.0/). The Open Database License (ODbL) is a license agreement intended to allow users to freely share, modify, and use this Dataset while maintaining this same freedom for others, provided that the original source and author(s) are credited. Yonglan Tuo, Minghao Liu, Zhengxiang Qi, Hang Chu, Jiajun Hu, Bo Zhang, Yu Li, Xiao Li
